# Low TSH and low T3 hormone levels as a prognostic for mortality in COVID-19 intensive care patients

**DOI:** 10.3389/fendo.2024.1322487

**Published:** 2024-04-25

**Authors:** Or Sror-Turkel, Nidal El-Khatib, Adi Sharabi-Nov, Yaniv Avraham, Shlomo Merchavy

**Affiliations:** ^1^ Azrieli Faculty of Medicine, Bar-Ilan University, Safed, Israel; ^2^ Department of ENT, Ziv Medical Center, Safed, Israel; ^3^ Statistics Unit, Ziv Medical Center, Safed, Israel; ^4^ Department of Statistics, Tel-Hai Academic College, Tel-Hai, Israel

**Keywords:** thyroid hormones, COVID-19, intensive care unit, mortality, TSH, T3

## Abstract

**Introduction:**

Coronavirus diasease 2019 (COVID-19) can cause both pulmonary and systemic inflammation, potentially determining multi-organ dysfunction. The thyroid gland is a neuroendocrine organ that plays an important role in regulating immunity and metabolism. Low serum levels of thyroid hormones are common in critical disease situations. The association between low thyroid hormone levels and mortality in COVID-19 intensive care patients has yet to be studied.

**Aim:**

The aim of this study is to compare thyroid hormone levels between patients in the general intensive care unit (ICU) to patients in the COVID-19 ICU.

**Methods:**

This was a retrospective comparative study of 210 patients who were hospitalized at Ziv Medical Center in the general ICU and in the COVID-19 ICU. Clinical and demographic data were collected from patient’s electronic medical records.

**Results:**

Serum thyroid hormone levels of Thyroid Simulating Hormone (TSH), T4, and T3 were significantly lower in COVID-19 intensive care unit patients compared to the patients from the general intensive care unit (p < 0.05). The mortality rate in the COVID-19 ICU (44.4%) was higher compared to that in the general ICU (27.3%) (p < 0.05). No significant statistical difference was observed between the two groups in terms of gender and recorded comorbidities of diabetes mellitus, cerebral vascular accident, kidney disease, and cancer.

**Conclusions:**

Low serum thyroid hormone levels—T3, T4, and TSH—in COVID-19 ICU patients are associated with higher mortality and could possibly be used as a prognostic factor for mortality among COVID-19 ICU patients. Thyroid hormone levels should be a part in the routine evaluation of COVID-19 ICU patients.

## Introduction

Coronavirus disease 2019 (COVID-19) is caused by severe acute respiratory syndrome coronavirus 2 (SARS-CoV-2). The disease has been considered a global epidemic that has caused a significant health burden. The clinical presentation of COVID-19 infection is wide and can range from asymptomatic to severe and fatal disease (1).

The physiologic outcome of a COVID-19 infection may be attributed to the virus’ unique features (2). COVID-19 can cause both pulmonary and systemic inflammation, potentially determining multi-organ dysfunction (3, 4). COVID-19 virus infects human tissues by entering cells via the angiotensin-converting enzyme 2 (ACE2) receptor (5, 6). The thyroid has a highly important role in regulating immunity and metabolism. Thyroid hormones and immunomodulatory molecules are involved in the complex interplay between the thyroid and viral infection response. Viral infections and the associated inflammatory and immune responses may have significant effects on thyroid function. The thyroid has high ACE2 tissue expression, which has been associated with immune signatures, making the thyroid a possible target of COVID-19 infection (7). Because thyroid hormones affect multiple organ systems, including the cardiovascular and respiratory systems, thyroid dysfunction may have a direct effect on the course of COVID-19 (8).

Recent studies have investigated the effect of COVID-19 disease severity and thyroid function (9–11).. Thyroid hormones influence both the innate and adaptive immune responses. In the course of a regular immune response, T4 and T3 increase the release of various cytokines that are part of the “cytokine storm” that might be induced by systemic viral infections. As a result, it is highly possible that the cytokine storm associated with severe COVID-19 infection may be the result of thyroid dysfunction indirectly through inflammatory mediation (12).

Several studies have shown a relationship between COVID-19 infection and subacute thyroiditis. It is widely recognized that subacute thyroiditis is caused by a viral infection or a post-viral inflammatory response. The dysregulation of the hypothalamus–pituitary–thyroid axis has also been considered, at least, in part, responsible for hypothyroidism in COVID-19 (13). Previous studies suggested that thyroid dysfunction is caused by direct infection of the thyroid or a “cytokine storm”–mediated autoimmune effect on the thyroid (8). The specific damage caused by COVID-19 to thyroid function remains to be fully clarified (5, 14).

Euthyroid sick syndrome (ESS), also known as non-thyroidal illness syndrome, is the physiologic adaptation and pathologic response to acute disease. ESS may also be present in intensive care unit (ICU) patients. ESS commonly presents with low T3 levels alongside normal T4 and TSH levels (6, 7, 15). Previous studies have shown that low serum levels of T3 are usually associated with disease severity and deterioration in critical illness, as well as ICU admission (7, 9, 11, 16).

A recent published study has shown that COVID-19 ICU patients had lower T3, T4, and TSH serum levels in comparison with COVID-19 non-ICU patients (17). In this study, we compared thyroid hormone levels between COVID-19 ICU patients to non-COVID-19 ICU patients in association to disease severity and mortality.

## Methods

### Participants

The study was approved by the health ethics committee of our institution in accordance of the declaration of Helsinki. This was a retrospective study including 210 patients who were hospitalized at our medical center in the general ICU or in the COVID-19 ICU. Of these, 45 patients were hospitalized in the designated COVID-19 ICU between December 2020 and October 2021. Patients included in this group were positive for SARS-CoV-2 by reverse transcriptase polymerase chain reaction assay. The control group included 165 patients hospitalized in the general ICU from September 2018 to December 2019, because, at that time period, COVID-19 was not present in this country. Patients with a history of any thyroid disorder and/or under 18 years old were excluded from the study.

### Measurements

Data were collected from patient files. The information collected included demographic data and clinical data—comorbidities, duration of hospitalization, mortality, and levels of serum thyroid hormones (T3, T4, and TSH). Measurement of thyroid hormones is a routine procedure upon admission to the ICU.

### Biochemical assays

The serum levels of T3, T4, and TSH were measured by electrochemiluminescence immunoassay “ECLIA” method using Cobas e immunoassay analyzer, using the following Roche Diagnostics GmbH kits: Ref. 11731360 for T3, 06437281 for T4, and 08429324 for TSH. For each kit, the normal expected values correspond to the 2.5th and 97.5th percentile. In our laboratory, the reference ranges for TSH, T4. and T3 were 0.27–4.20 mU/L, 12–22 μg/dL, and 1.3-3.1 ng/dL, respectively.

### Statistical analysis

Descriptive statistics: For categorical variables, summary tables were provided, giving sample size and relative frequencies. For continuous variables, summary tables were provide giving arithmetic mean (M) and 95% confidence interval (CI). Pearson’s chi-squared was applied for testing the correlations between the study groups for the categorical parameters. The independent sample t-tests were applied to measure the differences between the study groups for the continuous variables. P-value of 5% or less was considered statistically significant. The data were analyzed using the SPSS software version 28 (IBM).

## Results

Two hundred ten patients were included in the study. Forty-five patients hospitalized in the COVID-19 ICU had a mean age of 63.4 years, with 66.7% being male patients. The other 165 patients hospitalized in the general ICU had a mean age of 68.3 years, with 56.4% being male patients (**Table 1**). Most patients in general ICU (63.4%) were of Jewish compared to COVID-19 ICU, in which most patients (60.0%) were of non-Jewish (p < 0.001) (**Table 1**). More patients in the general ICU group had hypertension and cardiovascular disease in their medical history (65.5% and 61.2%, respectively) compared to the COVID-19 ICU (48.1% and 44.4%, respectively, p < 0.05). No significant differences were observed between the two groups according to diabetes mellitus, cerebral vascular accident (CVA), kidney disease, and cancer (**Table 1**).

**Table 1:** Demographic characteristic of the study patients stratified by the type of ICU (general vs. COVID-19).

Variables	General (n = 165)	COVID-19 (n = 45)	P
Age, year [mean (95% CI)]	68.3 (65.7–71.0)	63.4 (59.5–67.4)	0.077
Gender (n, %)			
Male	93, 56.4	30, 66.7	0.214
Female	72, 43.6	15, 33.3	
Ethnicity (n, %)			
Jewish	104, 63.4	18, 40.0	0.005
Other	60, 36.6	27, 60.0	
Diabetes mellitus (n, %)	55, 33.3	11, 24.4	0.255
CVA (n, %)	19, 11.5	6, 13.3	0.738
Hypertension (n, %)	108, 65.5	25, 48.1	0.025
CVD (n, %)	101, 61.2	20, 44.4	0.044
Kidney disease (n, %)	39, 23.6	13, 28.9	0.469
Cancer (n, %)	21, 12.7	3, 6.7	0.257

ICU, intensive care unit; CVD, cardiovascular disease; CI, confidence interval.

The serum levels of TSH, T3, and T4 were all significantly lower in the COVID-19 ICU compared to that in the general ICU: TSH mean serum levels were 0.76 mU/L vs. 2.19 mU/L, respectively, p < 0.001; T3 mean serum levels were 0.89 ng/dL vs. 1.17 ng/dL, respectively, p < 0.01; and T4 mean serum levels were 14.77 µg/dL vs. 15.90 μg/dL, respectively, p < 0.05 (**Table 2**). The mortality rate in the COVID-19 ICU was significantly higher compared to the general ICU (44.4% vs. 27.3% respectively, p < 0.05) (**Table 2**). The duration of hospitalization in the general ICU was longer compared to that in the COVID-19 ICU, but with no statistical significance (**Table 2**).

**Table 2:** Clinical characteristic of the study patients stratified by the type of ICU (general vs. COVID-19).

Variables	General (n = 165)	COVID-19 (n = 45)	P
Days in ICU [mean (95% CI)]	7.7 (5.9–9.4)	6.1 (4.4–7.9)	0.544
Days in hospital [mean (95% CI)]	24.6 (20.3–28.9)	20.3 (16.6–24.1)	0.294
TSH [mean (95% CI)]	2.19 (1.78–2.60)	0.76 (0.47–1.05)	<0.001
T_3_ [mean (95% CI)]	1.17 (1.11–1.24)	0.89 (0.81–0.97)	<0.001
T_4_ [mean (95% CI)]	15.90 (15.34–16.46)	14.77 (13.53–16.02)	0.045
Last status (n, %)			
Alive	120, 72.7	25, 55.6	0.027
Dead	45, 27.3	20, 44.4	

The mortality frequency in the COVID-19 ICU was significantly associated with all low serum thyroid hormones level, with low T3 being most dominant (**Table 3**).

**Table 3:** Distribution of patients with low serum levels (%) of T3, T4, and TSH stratified by the type of ICU and according to their last status.

Variables (normal range)	General (n = 165)		COVID-19 (n = 45)		p
	Alive (n = 120)	Dead (n = 45)	Alive (n = 25)	Dead (n = 20)	
	Low serum levels (n, %)*				
T_3_ (1.3–3.1 ng/dL)	75, 63.6c	32, 71.1bc	20, 87.0ab	20, 100a	0.002
T_4_ (12–22 μg/dL)	9, 7.5b	10, 22.2a	2, 8.7b	9, 45.0a	<0.001
TSH (0.27–4.20 mU/L)	6, 5.0c	10, 22.2b	8, 32.0ab	11, 55.0a	<0.001

*a–c, different letters in each row represent significant differences between the frequencies.

## Discussion

Our study is the first to compare thyroid hormone serum levels in critical COVID-19 patients to non–COVID-19 critical patients. Serum thyroid hormone levels of TSH, T3, and T4 were significantly lower in the COVID-19 ICU compared to that in the general ICU (**Table 2**). At the same time, the mortality rate in the COVID-19 ICU was higher compared to that in the general ICU (p < 0.05) (**Table 2**). Our result show that low serum thyroid hormone levels—T3, T4, and TSH—in COVID-19 ICU patients are associated with higher mortality and could possibly be used as a prognostic factor for mortality among COVID-19 ICU patients (**Table 3**).

Changes in thyroid hormones levels and especially low T3 are usually correlated with a prognosis of deterioration in sever disease and may result in longer hospitalization duration and ICU admission (7, 16). Three major types of thyroid dysfunction caused by COVID-19 have been identified: mild to moderate ESS, severe ESS, and subclinical hypothyroidism. These three types of thyroid dysfunction were associated with the severity of COVID-19 prognosis in previous studies (18). The clinical picture observed in the COVID-19 ICU patients, with all low T3, T4, and TSH serum levels, may support the theory of COVID-19 damage to the hypothalamus–pituitary–thyroid axis. The direct cytotoxic effects of the virus on the pituitary gland and its indirect effects through of pro-inflammatory activation of cytokine production create a “cytokine storm” that, in turn, induces ESS, leading to selective transient pituitary dysregulation, which affects thyroid function (18). This mechanism has been described in previous publications (19). A study by Campi et al. (2021) found that low TSH levels were present at admission or hospitalization in 39% of COVID-19 patients and were associated with low T3 in 50% of patients. They concluded that the observed thyroid dysfunction is probably from the cytokine storm induced by SARS-CoV-2 and not a destructive thyroiditis (20). Lania et al. (2020) also found that thyroid dysfunction was associated with high levels of IL-6 in patients with SARS-CoV-2 infection (12). Recent published studies from 2023 also support this theory, describing lower levels of T3 and TSH associated with significantly higher blood levels of inflammatory markers in severe COVID-19 patients (21). This may serve as a possible explanation for the high mortality rate observed in the COVID-19 ICU in comparison to that in the general ICU. The exact mechanisms by which a COVID-19 associated cytokine storm induces low thyroid hormone levels and the effect of low thyroid hormones level on COVID-19 course of disease are still unclear and need further study.

The possibility of thyroid dysfunction lasting beyond the acute phase of COVID-19 was not tested in this study due to loss of follow-up of most patients. The immunization status of patients was not included because, prior to 2020, COVID-19 vaccination did not exist, and, in the COVID-19 group, data were not available for all study participants.

The longer hospitalization duration of patients in the general ICU might be explained by the fact that the patients in the general ICU were of older age, had more underlying comorbidities (**Table 1**), and possibly also due to the higher mortality rate in the COVID-19 ICU (**Table 3**) (16). The statistical differences of ethnicity between the patient groups (**Table 1**) can be explained by the population composition of the medical center vicinity. More patients in the general ICU group had hypertension and CVD in their medical history (65.5% and 61.2%, respectively) compared to the COVID-19 ICU (48.1% and 44.4%, respectively, p < 0.05). The possible influence of these factors on thyroid function is not known and has not been studied yet.

## Conclusions

Low T3, T4, and TSH serum levels are associated with higher mortality in COVID-19 ICU patients. This can possibly be considered as a negative prognostic factor for COVID-19 ICU patients. COVID-19 infection may damage thyroid functions. Therefore, thyroid hormone levels should be a part in the routine evaluation of COVID-19 patients. Further studies of thyroid functions in post COVID-19 patients are warranted.

## Data availability statement

The raw data supporting the conclusions of this article will be made available by the authors, without undue reservation.

## Ethics statement

The studies involving humans were approved by Ziv Medical Center ethics committee. The studies were conducted in accordance with the local legislation and institutional requirements. The human samples used in this study were acquired from a by- product of routine care or industry. Written informed consent for participation was not required from the participants or the participants’ legal guardians/next of kin in accordance with the national legislation and institutional requirements.

## Author contributions

OS-T: Data curation, Investigation, Writing – review & editing. YA: Conceptualization, Methodology, Writing – original draft, Writing – review & editing. AS-N: Data curation, Formal analysis, Software, Writing – review & editing. NE-K: Data curation, Investigation, Writing – review & editing. SM: Conceptualization, Project administration, Supervision, Writing – review & editing.
